# Study of wound healing in rats treated with topical and injected mitomycin-C

**DOI:** 10.1016/S1808-8694(15)30563-2

**Published:** 2015-10-19

**Authors:** Fernando de Andrade Quintanilha Ribeiro, Janaína de Pádua Borges, Lusiele Guaraldo, Maria Regina Vianna

**Affiliations:** 1 Associate Professor - Otorhinolaryngology Department at the Faculdade de Ciências Médicas da Santa Casa de São Paulo.; 2 MD, MSc (Pharmacology Department, University of Sao Paulo), Pharmacist with the surgery team at the Faculdade de Ciências Médicas da Santa Casa de São Paulo.; 3 Associate Professor - Department of Physiologic Sciences at the Faculdade de Ciências Médicas da Santa Casa de São Paulo.; 4 Professor, PhD (University of São Paulo). Pathologist at the Alemão Oswaldo Cruz Hospital. Otorhinolaryngologist, Otorhinolaryngology Department at the Faculdade de Ciências Médicas da Santa Casa de São Paulo.

**Keywords:** wound healing, topical, mitomycin-C, rats

## Abstract

Experimental study in animals. Introduction: Mitomycin C has been used as a fibroblasts inhibitor, thus reducing the scaring process in surgical wounds.

**Aim:**

This paper aims at assessing the use of Mitomycin C to reduce the scaring process through its topical use and later injected reinforcements.

**Materials and Methods:**

We used a model of a creating a wound in the dorsum of rats, removing a circular piece of skin and letting it heal by itself. We used 18 rats, divided in three groups. The first group - Control, the second with topical use, and a third group with injected mitomycin C reinforcement, monthly for 2 months. After 3 months the animals were slaughtered and the scars were surgically removed and sent for histology study.

**Results:**

We noticed, under different criteria, that healing with topical mitomycin is less intense; however, when it was injected, the parameters were again comparable to those from the control group.

**Discussion:**

We believe that injected mitomycin C in the scar, since it is highly toxic, it destroys tissue and brings about scar neoformation.

**Conclusions:**

Mitomycin C reduces the scaring process when used topically; however, it increases scar tissue formation when injected in these wounds.

## INTRODUCTION

Mitomycin-C has been broadly used as a chemotherapeutic agent^1^. Lately, however, its role as a fibroblast inhibitor^2^ in delaying the healing process has been explored in surgical procedures where sequelae such as stenosis and adhesion may occur. A number of papers in the literature have shown such effect in surgical settings, many of which in otorhinolaryngology^3^, ^4^, ^5^, ^6^, ^7^. Studies with animal models have also been conducted^8^, ^9^, but consensus has not been reached and contradictory evidences question its true efficacy. Many papers have shown that topical mitomycin-C alone might fail to achieve the desired results^10^. Injections of the agent have therefore been added to strengthen its effectiveness and to further mitigate the occurrence of surgical fibrosis^11^, ^12^, ^13^.

## OBJECTIVE

This paper aims to compare the responses of rat wound models where mitomycin-C was applied topically alone or topically in conjunction with monthly reinforcement injections.

## MATERIALS AND METHODS

This paper was approved by the animal studies ethics committee of the institution, under permit # 10 of May 14, 2002. Eighteen young male Wistar rats weighing between 355 and 400 grams were used. The animals were kept in separate cages in the lab of the Surgical Technique Unit of the institution. The rats were anesthetized with Ketamine (Francotar®) 10ml in concentrations of 100mg/ml and doses of 0.5 ml/rat and Xylazine (Rompun®) 10ml in concentrations of 20mg/ml and doses of 0.1ml/rat. While under general anesthesia, the rats had identical wounds of two centimeters in diameter made in their dorsa. All eighteen rats were numbered and divided into three groups of six subjects each. The control group had their wounds treated topically with gauze soaked in saline solution for five minutes. The wounds of the remaining two groups were treated with gauze soaked in one milliliter of mitomycin-C in concentrations of 0.5ml/mg for five minutes and then washed with 10 ml of saline solution. Every thirty days - twice in all - the subjects on group three were injected a 0.01mg/ml solution of mitomycin-C into their scars. Equal volumes of saline solution were injected in the subjects of the two remaining groups. The adopted topical concentration was based in the values reported in the literature^10^, ^13^. A previous paper indicated that the injection concentration used in this study would not result in rat tissue necrosis^11^. Two rats in the control group died as they were being anesthetized. All rats were slaughtered after 90 days (intracardiac injection of 19.1% KCl after general anesthesia), their scars surgically removed and sent for histology. The specimens were randomly numbered and sent to the pathologist who examined, embedded, sliced, and stained them with H&E. The slides were assessed in terms of the degree of fibrosis, vascular proliferation, number of fibroblasts and fibrocytes.

## RESULTS

**Chart 1:**
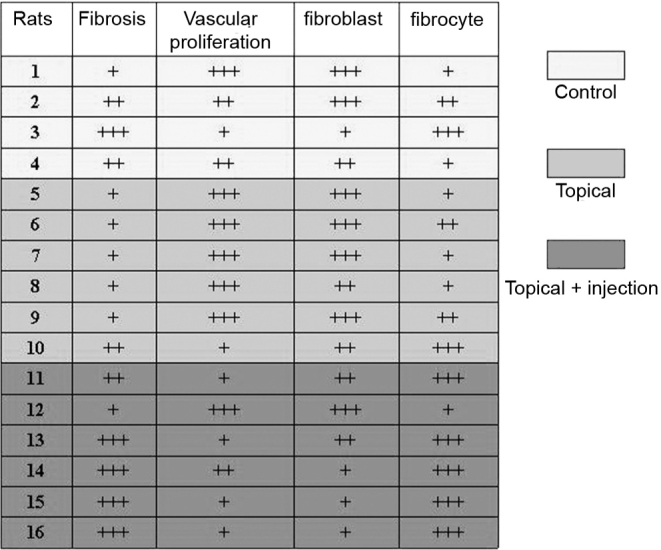
Rat wound assessment in terms of fibrosis, vascular proliferation, number of fibroblasts, and number of fibrocytes.

The slides were analyzed and each parameter was given a +, ++ or +++ rating, depending on the intensity of the outcome. The results can be seen in Chart 1.

## DISCUSSION

### Degree of fibrosis

Scar tissue intensity may be characterized by the degree of fibrosis present in it.

### Vascular proliferation

It is more present in less defined or evolved scar tissue.

### Number of fibroblasts

The greater the number of fibroblasts, the less mature and established is the healing process.

### Number of fibrocytes

The greater the number of fibrocytes, the older and more intense is the scar.

As seen in the chart, the subjects in group 2 - topical mitomycin-C alone - have presented the least intense and developed scar tissues. Group 3 - topical and injected mitomycin-C - was expected to have an even more incipient formation of scar tissue, as its subjects were given an additional shot of mitomycin-C every thirty days, but healing was nonetheless more intense. This is possibly due to the high toxicity and necrotizing characteristics of mitomycin-C. Therefore, every thirty days the healing tissue would be attacked, which led the healing process to start all over again. Perhaps the already quite low concentration of 0.01ml/mg could be further reduced to yield better results. The mechanical impact of the injection was disregarded, as all other groups were given the same number of shots and same volume of saline solution.

The low number of subjects rendered the statistical assessment attempts impractical. The + rating system was thus also adopted for this parameter. The ratings for each rat were given based on the parameter assessed in relation to the healing degree. Pluses (+) were given to fibrosis and fibrocytes scores and minuses (-) to vascular proliferation and fibroblasts. Below are the scores for each of the subjects:

1: +1-3-3+1= - 4.
2: +2-2-3+2=-1
3: +3-1-1+3= + 4
4: +2-2-2+1= - 1
5: +1-3-3+1= - 4
6: +1-3-3+2= - 3
7: +1-3-3+1= - 4
8: +1-3-2+1= - 3
9: +1-3-3+2= - 3
10: +2-1-1+3= + 3
11: +2-1-2+3= + 2
12: +1-3-3+1= - 4
13: +3-1-2+3= + 3
14: +3-2-1+3= + 3
15: +3-1-1+3=+ 4
16: +3-1-1+3= + 4

The summation of the scores for subjects one to four (control group) was -2.

The summation of the scores for subjects five to ten (topical) was -14.

The summation of the scores for subjects eleven to sixteen (topical + injection) was +12.

After removing the most discrepant subjects from the groups (number 3 from the control group, number 10 from the topical treatment group, and number 12 from the topical treatment + injection group), the results were as follows:

6 Control group: -6
17 Topical treatment group: -17
16 Topical treatment + injection: +16

Thus, considering that healing can be assessed as more intense when more fibrosis and fibrocytes are present (+) and less intense when more vascular proliferation and fibroblasts are present (-), it was realized that after 90 days of treatment the group using only topical mitomycin-C had comparatively less healing than the control group, while the group under the topical treatment and injection regimen had much more intense scar tissue formation.

One should notice that the large number of fibroblasts and extensive vascular proliferation found in the topical mitomycin-C group after 90 days of treatment has yielded laxer and more fragile scar tissues. A longer term study is thus necessary to verify whether such trait will persist.

## CONCLUSIONS

After 90 days of treatment, mitomycin-C was shown to be more effective in controlling the healing process when used only topically. When injected every 30 days for the duration of the experiment, healing was more intense than observed in the control group.

## References

[1] Bradner WT (2001). Mitomycin C: a clinical update. Cancer Treat Rev.

[2] Hu D, Sires BS, Tong DC, Royack GA, Oda D (2000). Effect of brief exposure to mitomycin C on cultured human nasal mucosa fibroblasts:. Ophthal Plast Reconstr Surg.

[3] Ingrams DR, Volk MS, Biesman BS, Pankratov MM, Shapshay SM (1998). Sinus surgery: does mitomicyn-C reduce stenosis?. Laryngoscope.

[4] Spector J E, Werkhaven J A, Spector N C, Huang S, Page RN, Baranowski B, Luther M, McGehee B, Reinisch L (1999). Preservation of function and histologic appearance in the injured glottis with topical mitomycin C. Laryngoscope.

[5] Jassir D, Buchman CA, Gomez-Marin O (2001). Safety and efficacy of topical mitomycin C in myringotomy patency. Otolaryngol Head Neck Surg.

[6] Estrem SA, Batra PS (1999). Preventing myringotomy closure with topical Mitomicin C in rats. Otolaryngol Head Neck Surg.

[7] Yazawa Y, Suzuki M, Kitano H, Kitajima K (1999). Intraoperative mitomicyn-C in endolynphatic sac surgery for Ménière's disease: a pilot study:. ORL J Otorhinolaryngol Relat Spec.

[8] Sampaio MW, José NK, Alves MR (1995). Efeitos do uso tópico da Mitomicina C em olhos de ratas. Arq Bras Oftalmol.

[9] Mattar DB, Alves MR, Silva MHT, José NK (1995). Estudo da influência da aplicação subconjuntival da Mitomicina C na reparação de defeito epitelial corneano, em coelhas. Arq Bras Oftalmol.

[10] Ribeiro FAQ, Guaraldo L, Borges JP, Zacchi FFS, Eckley CA (2004). Clinical and Histological Healing of Surgical Wounds Treated with Mitomicyn C. Laryngoscope.

[11] Ribeiro FAQ, Borges JP, Zacchi FFS, Guaraldo L (2003). O Comportamento clínico e histológico da pele do rato submetida ao uso tópico e injetável de Mitomicina C. Rev Bras Otorrinolaringol.

[12] Ferguson B, Gray SD, Thibeault S (2005). Time and dose effects of mitomycin C on extracellular matrix fibroblasts and proteins. Laryngoscope.

[13] Gray SD, Tritle N, Li W (2003). The effect of mitomycin on extracellular matrix proteins in a rat wound model. Laryngoscope.

